# Investigating Health Inequality Using Trend, Decomposition and Spatial Analyses: A Study of Maternal Health Service Use in Nepal

**DOI:** 10.3389/ijph.2023.1605457

**Published:** 2023-06-02

**Authors:** Shehzad Ali, Amardeep Thind, Saverio Stranges, M. Karen Campbell, Ishor Sharma

**Affiliations:** ^1^ Department of Epidemiology and Biostatistics, Western University, London, ON, Canada; ^2^ Interfaculty Program in Public Health, Western University, London, ON, Canada; ^3^ WHO Collaborating Centre for KT and HTA in Health Equity, Ottawa, ON, Canada; ^4^ Department of Health Sciences, University of York, York, United Kingdom; ^5^ Department of Psychology, Macquarie University, Sydney, NSW, Australia; ^6^ Departments of Family Medicine and Medicine, Schulich School of Medicine & Dentistry, Western University, London, ON, Canada; ^7^ Department of Precision Health, Luxembourg Institute of Health, Strassen, Luxembourg; ^8^ Department of Pediatrics, Schulich School of Medicine & Dentistry, Western University, London, ON, Canada; ^9^ Department of Obstetrics & Gynecology, Schulich School of Medicine & Dentistry, Western University, London, ON, Canada; ^10^ Children’s Health Research Institute, Lawson Health Research Institute, London, ON, Canada

**Keywords:** health inequality, equity, decomposition analysis, spatial analysis, maternal health services, antenatal, postnatal, Nepal

## Abstract

**Objectives:** (a) To quantify the level and changes in socioeconomic inequality in the utilization of antenatal care (ANC), institutional delivery (ID) and postnatal care (PNC) in Nepal over a 20-year period; (b) identify key drivers of inequality using decomposition analysis; and (c) identify geographical clusters with low service utilization to inform policy.

**Methods:** Data from the most recent five waves of the Demographic Health Survey were used. All outcomes were defined as binary variables: ANC (=1 if ≥4 visits), ID (=1 if place of delivery was a public or private healthcare facility), and PNC (=1 if ≥1 visits). Indices of inequality were computed at national and provincial-level. Inequality was decomposed into explanatory components using Fairile decomposition. Spatial maps identified clusters of low service utilization.

**Results:** During 1996–2016, socioeconomic inequality in ANC and ID reduced by 10 and 23 percentage points, respectively. For PND, the gap remained unchanged at 40 percentage points. Parity, maternal education, and travel time to health facility were the key drivers of inequality. Clusters of low utilization were displayed on spatial maps, alongside deprivation and travel time to health facility.

**Conclusion:** Inequalities in the utilization of ANC, ID and PNC are significant and persistent. Interventions targeting maternal education and distance to health facilities can significantly reduce the gap.

## Introduction

Maternal and neonatal health is a major public health concern for Nepal [1]which has an estimated maternal mortality rate (MMR) of 239 per 100,000 live births and neonatal mortality rate (NMR) of 21 per 1,000 live births [2]. These rates are well above the global average of 152 deaths per 100,000 live births and 17 deaths per 1,000 live births, respectively, for MMR and NMR [1, 3–5]. Also, there exists a significant socioeconomic gradient in these outcomes which are worse in poorest quintiles [5, 6]. Nepal aims to significantly reduce these rates by 2030 to meet the Sustainable Development Goals (SDGs) of <70 per 100,000 live births for MMR and <12 per 1,000 live births for NMR [7].

Most maternal and neonatal morbidity and mortality is preventable, or at least manageable through timely intervention during the antenatal, delivery and postnatal periods [8–12]. Antenatal care (ANC) is important for early detection and management of high-risk pregnancies and has been effective in reducing NMR and MMR globally [13–19]. Following the antenatal period, institutional delivery (ID) (defined as birth in a health facility) is associated with significant reduction in maternal and neonatal deaths [20–24]. After delivery, almost 60% of all maternal deaths and 3/4th of all neonatal deaths occur during the post-partum period [25–27] which can be significantly reduced by timely access to postnatal care (PNC) [10, 28]. In addition, PNC provides an opportunity for health promotion, nutrition supplementation, immunization, breast-feeding counselling, feeding practices and family planning [9].

The World Health Organization (WHO) recommends at least four antenatal visits to a skilled health attendant [9, 29]. For ID, it recommends that all childbirths take place at a health facility staffed by trained birth attendants [22]. Following birth, in the case of ID, an initial postnatal appointment with a trained birth attendant should happen within the first hour and then again before discharge. In the case of home delivery, the mother should be visited within 24 h of the birth. Follow-up postnatal visits to assess the health of mother and infant are recommended at 2–3 days, 6–7 days and at week 6 for both institutional and home delivery [30].

In Nepal, while utilization of ANC, ID and PNC (hereon referred to as “maternal health services”) has improved over the last 20 years, these rates remain well below the global average [31]. Compared to its South Asian neighbours, Nepal has much lower utilization rates than India, Sri Lanka, Bhutan and Maldives, and only marginally better than Pakistan, Afghanistan and Bangladesh [32]. Almost 32% of pregnant women in Nepal do not receive the recommended number of ANC visits, 40% of the deliveries occur at home and almost the same percentage do not receive any PNC [2]. In addition, socioeconomic inequality in utilization of maternal health services has been reported in the literature [33, 34]. Compared to those in the poorest quintile, women in the richest quintile have higher probability of having ANC visits (by 27%), PNC visits (by 42%) and ID (by 56%) [2, 35]. Nepal also has significant regional variation in utilization of maternal health services, with much lower levels of access in rural and remote regions of the country [36, 37]. This is due to poor availability of healthcare services, poor parental education, cultural beliefs and difficult geographic terrain [19, 38–43].

A few of studies have investigated inequalities in the use of maternal health services in Nepal. Sapkota et al [34] used data from Demographic and Health Survey (DHS) and found that socioeconomic-related inequality in the use of ANC and ID has reduced over time, both at national and provincial levels, except in the Karnali province [34]. These findings were consistent with Mehata et al [44] who observed a comparable decrease in socioeconomic inequality in ANC and ID at the national-level between 1996 and 2011 [44]. Arsenault et al [31] compared inequality in ANC visits across 91 low- and middle-income countries, including Nepal, using the most recent (single) wave of DHS and Multiple Indicator Cluster Survey (MICS) data; their study found that Nepal had one of the highest values of slope and relative indices on inequality [31].

Further studies have investigated the overall determinants of maternal health service use in Nepal, without investigating inequalities. For instance, Khatri et al [45], conducted a cross-sectional analysis of DHS 2016 data and found that, in general, women with higher wealth status, higher education level and socially-advantaged ethnicity had higher odds of receiving maternal health services [45]. Other studies focused on one of the three services (ANC, ID or PNC). For ANC visits, in addition to the above factors [45, 46], greater participation in household decision-making and migrant status [47] were associated with higher odds of service use, while higher parity and rural residence were associated with lower odds [37]. For ID use, attendance of ANC services and residence in urban areas were associated with higher odds while older age was associated with lower odds [48, 49]. Finally, for PNC services, the use of ANC visits, place of delivery and maternal occupation and autonomy were found to be important determinants [50, 51]. In addition, travel time to the nearest health facility was identified as an important barrier to accessing maternal health services [50].

While the above studies provide some insight into inequalities, there is limited evidence on the contribution of social and geographical determinants towards inequality across all three maternal health services (ANC, ID and PNC) and the spatial distribution of service use at local administrative level to inform decision making. To address the current knowledge gap, our study aims to: 1) evaluate the level and changes in the utilization of ANC, ID and PNC over a 20-year period; 2) estimate the slope and relative socioeconomic inequality in utilization of these services over time; and 3) identify the key drivers of socioeconomic inequality by conducting a decomposition analysis using the most recent year of data. In addition, to inform policy decisions, our study has two additional objectives: 4) to understand how geographical areas (clusters) vary in terms of utilization of ANC, ID and PNC; and 5) to spatially display cluster-level service utilization in relation to socioeconomic status and travel time to the nearest health facility. This will provide policy-relevant evidence to address socioeconomic inequality in Nepal.

## Methods

### Design and Setting of the Study

This study used data from the most recent five waves of the National Demographic and Health Survey (DHS), conducted in 1996, 2001, 2006, 2011 and 2016. DHS is a cross-sectional survey that uses a multi-stage stratified cluster sampling technique. First, wards are selected as primary sampling units (PSU), and then one enumeration area (EA) is selected within each ward, followed by a sampling of 30 households from each EA [2]. For the current study, women aged 15–49 who had a live birth in the past 5 years are included in the analysis. Since Nepal’s current provincial structure was first established in 2015, all provincial-level analyses used only the most recent wave of DHS (i.e., 2016).

The outcome was defined based on the DHS working paper (DHS 7) for the most recent birth (midx = 1; variable names are based on the DHS dataset) [52]. ANC is defined as a binary outcome (0/1), i.e., receiving four or more visits during pregnancy (m14>3). A childbirth is classed as ID (=yes) if the place of delivery (m15) was either a public healthcare facility (i.e., a government hospital, primary healthcare centre, health post, sub-health post or other public sector) or private health facility (i.e., private hospital, clinic, nursing home, non-governmental organization-run health centre or other private health facilities). PNC (m62; m66) is defined as a binary outcome (0/1) based on having at least one visit to the facility where the child was born or at home. The first two waves of the DHS survey did not record postnatal visits at the place of delivery [45]; hence, data from 2006–2016 were used for the PNC outcome.

Socioeconomic status (SES) was defined based on the household wealth index (HWI); this is a composite measure of household living standard and is commonly used as a proxy measure of SES [53]. HWI is constructed using principal component analysis (PCA) based on household assets [54]. Based on HWI, households are classified into quintiles, with the first quintile (Q1) being the poorest group and the fifth quintile (Q5) being the richest group.

### Socioeconomic Gap and Inequality Indices (1996–2016)

First, a descriptive analysis was conducted to assess the level and changes in the utilization of ANC, ID and PNC services across years using wave-specific sampling weights. We then investigated how service utilization varied between socioeconomic quintiles. Next, socioeconomic inequality in service use was quantified using the slope index of inequality (SII) and the relative index of inequality (RII) [55]. SII was estimated by assigning a fractional rank to each respondent based on their HWI, such that the richest household had a score of 1 and the poorest had a score of 0. This fractional rank is then used as a covariate in the logistic regression with ANC, ID, and PNC as separate outcome variables. Marginal probabilities were then used to estimate the absolute gap in utilization of the three maternal health services between the two ends of the wealth ranks. SII was divided by the average utilization proportion of these indicators to assess the relative measure of inequality (RII) [56].

### Spatial Distribution of Maternal Health Service Utilization (2016)

Geographic variation in the use of maternal health services, both between and within provinces was investigated, using spatial maps developed in ArcMap 10.7. We used data from the most recent wave (2016) as it represents the current provincial structure and local administrative units (or “clusters”) to visually display variation in utilization of all three services. These maps are useful for decision-makers as they pinpoint geographical clusters with low levels of utilization of services. We then used spatial maps to assess if cluster deprivation (computed as the mean HWI of the cluster) was associated with the level of ANC, ID and PNC utilization. This would help understand if poorer clusters had lower levels of service use than wealthier clusters.

### Decomposition Analysis (2016)

Fairlie Decomposition was used to identify the factors explaining the socioeconomic gap in the utilization of ANC, ID and PNC [57]. This approach partitions the socioeconomic gap into explained and unexplained portions. It generates simulated samples of data that pair observations (one-to-one matching) from the richest and poorest socioeconomic quintiles and estimates the predicted differences between those samples. To implement this, a previously published SAS code was used to generate random subsamples from the poorest quintile (equal to the size of the richest quintile) with randomly ordered variables; 1,000 replications were used to decompose the contributions of each independent variables to the socioeconomic gap [58, 59]. Mathematically, the average difference in the use of maternal health services (ANC/ID/PNC) between the richest and poorest quintiles can be expressed as:

$$\begin{array}{c} {\bar{Y}}^{lw}-{\bar{Y}}^{hw}=\left\lceil \sum_{i=1}^{N^{lw}}\frac{F\;\left(x_{i}^{lw}\;{\hat{\beta }}^{lw}\right)}{N^{lw}}-\sum_{i=1}^{N^{hw}}\frac{F\left(x_{i}^{hw}\;{\hat{\beta }}^{lw}\right)}{N^{hw}}\right\rceil \\ +\left\lceil \sum_{i=1}^{N^{hw}}\frac{F\;\left(x_{i}^{hw}\;{\hat{\beta }}^{lw}\right)}{N^{hw}}-\sum_{i=1}^{N^{hw}}\frac{F\left(x_{i}^{hw}\;{\hat{\beta }}^{hw}\right)}{N^{hw}}\right\rceil \end{array}$$

where, *Y ®* is the average probability of utilization of ANC, ID, and PNC. “*lw*” represents the lowest (poorest) socioeconomic quintile and “*hw*” the highest (richest) socioeconomic quintile. “*X*” represents a vector of independent variables, β̂ is a vector of coefficients estimated separately for each of the two groups using the pooled sample, *N* is the number of observations in a given group, and *F* is the cumulative logistic distribution function. Six independent variables were included in the analysis, based on the literature and data availability. These included: parity (1, 2 and 3+), age of the mother (<20, 20–30, 30+), mother’s education (no formal education/educated), father’s education (no formal education/educated), travel time (in minutes) to the nearest health facility and caste/ethnicity (Brahmins and Chhetri as upper social caste, and Janjati, Dalit and others considered lower social caste).

## Results

### Characteristics of the Study Population

Between 1996 and 2016, the mean age at delivery has remained relatively stable at 19 years (Table 1). During this period, the percentage of mothers with at least school-level education increased from 20.4% in 1996 to 69.8% in 2016, and the percentage of households with a female head increased from 7.7% in 1996 to 31.5% in 2016. There was also a sharp increase in the percentage reporting urban residence, from 8.9% in 1996 to 58.3% in 2016. This is partly due to a change in administrative classification, as Nepal moved to a federal system and classified many rural areas as “Nagarpalika” (urban). The time to reach the nearest health facility was only recorded in 2016 and averaged walking time 40 min.

**TABLE 1:** Characteristics of the study population included in Nepal Demographic Health survey Nepal, 1996–2016.

	1996	2001	2006	2011	2016
Antenatal care (%) (95% CI)	9.31 (8.4, 10.17)	14.52 (13.52, 15.53)	28.94 (27.57, 30.32)	52.73 (51.19, 54.24)	67.77 (68.35, 71.19)
Institutional Delivery (%) (95% CI)	7.89 (7.09, 8.69)	8.87 (8.06,9.68)	17.56 (16.4, 18.7)	39.59 (38.09, 41.09)	57.36 (55.83, 58.90)
Postnatal care^a^ (%) (95% CI)	—	—	29.43 (27.39, 31.47)	45.96 (44.13, 47.8)	56.69 (54.38, 59.0)
Age at first delivery (years)	19.15 ± 3.01	19.31 ± 2.95	19.42 ± 3.1	19.76 ± 3.2	19.91 ± 3.32
Time to reach health facility (minutes)	—	—	—	—	39.52 ± 39.8
Mother had no education (%) (95% CI)	79.54 (78.3, 80.7)	72.33 (71.05, 73.60)	58.69 (57.20, 60.19)	43.27 (41.7, 44.7)	30.18 (29.30, 32.16)
Father had no education (%) (95% CI)	37.86 (36.4, 39.31)	33.52 (32.17, 34.86)	22.79 (21.5, 24.10)	18.26 (17.08, 19.45)	12.7 (11.6, 13.7)
Parity (number of children)	Median: 3	Median: 3	Median: 2	Median: 2	Median: 2
	Mean: 3.4 ± 2.2	Mean: 3.29 ± 2.2	Mean: 2.95 ± 2.01	Mean: 2.65 ± 1.82	Mean: 2.3 ± 1.5
Caste/ethnicity (advantageous group^b^)	—	—	39.08 (37.6, 40.6)	38.05 (36.56, 39.56)	33.97 (32.51, 35.44)
Sample size	4,320	4,723	4,181	4,079	4,006

^a^ Before 2003, women who gave birth in a health facility were excluded from this indicator based on the assumption that all women who deliver in a health facility receive post-natal care. In 2003, this was revised, and this indicator included all deliveries regardless of place of birth. For consistency, we did not use the first two waves for this indicator.

^b^ Advantageous groups included Hill Brahmin/Chettri, Terai Brahmin/Terai Chhetri. 2001 and 1996 DHS used different classification for ethnicity.

### Socioeconomic Gap and Inequality Indices for Utilization of Maternal Health Services

**FIGURE 1:**
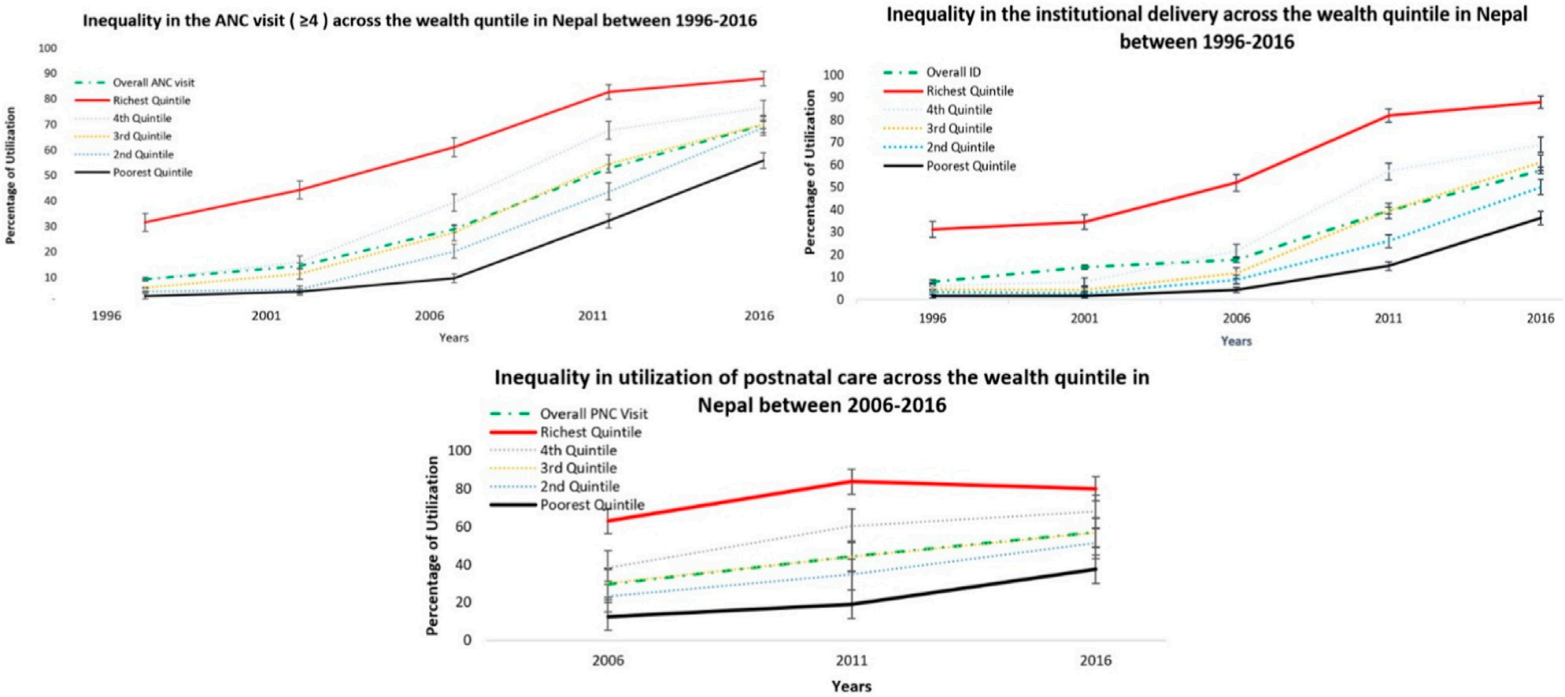
Rate of utilization of antenatal care, institutional delivery, and postnatal care, based on quintiles of socioeconomic status, Nepal 1996–2016.

On average, there has been a significant increase in the utilization of maternal health services in the last 20 years in Nepal (Table 1). The overall rate of ANC and ID service use has increased, respectively, from 9.3% to 7.9% in 1996 to 67.8% and 57.3% in 2016. The use of PNC, recorded from 2006, increased from 29.4% to 56.7% over 10 years. This increase in service use was observed across all socioeconomic groups, albeit at different rates (Figure 1). As a result, the gap between the richest and poorest quintiles in the use of ANC reduced from 29 percentage points in 1996 to 19 percentage points in 2016. For ID, the gap between socioeconomic groups increased over time from 29 percentage points in 1996 to 52 percentage points in 2016. This is largely due to much smaller increase in utilization in the poorest quintile. For PNC the socioeconomic gap was 40 percentage points in 2016 which has remained largely unchanged over time (Figure 1).

The SII for ANC and ID increased between 1996 and 2011 followed by a drop in 2016 (Table 2). This is because the increase in utilization in the first three waves was much larger in the richest quintile compared to the poorest quintile. SII for PNC was highest in 2006, followed by a reduction in subsequent waves. The RII has been falling for all three services over the study period (except for a small increase for ID services in 2001). This is because of a significant increase in average utilization of services (i.e., the denominator in RII) across all groups over time; as a result, the gap between the top and bottom quintiles represents a smaller fraction of the average utilization.

**TABLE 2:** Measure of Inequality in the utilization of maternal health services in Nepal 1996–2016.

	1996	2001	2006	2011	2016
Slope Index of Inequality (95% CI)					
ANC	32.1 (31.3, 34.0)	44.4 (43.4, 45.3)	57.9 (57.2, 58.6)	60.5 (60.2, 60.8)	36.3 (36.0, 36.5)
ID	33.7 (32.3, 34.3)	39.3 (38.2, 40.5)	53.1 (52.0, 54.0)	73.5 (72.8, 74.2)	56.6 (56.3, 56.9)
PNC	—	—	59.9 (58.9, 60.9)	47.6 (47.4, 47.9)	48.9 (48.6, 49.2)
Relative Index of Inequality (95% CI)					
ANC	3.5 (3.4, 3.7)	3.1 (3.1, 3.2)	2.0 (1.9, 2.1)	1.14 (1.13, 1.15)	0.52 (0.52, 0.53)
ID	4.3 (4.2, 4.4)	4.5 (4.3, 4.6)	2.0 (2.9, 3.1)	1.8 (1.8, 1.8)	0.9 (0.8, 0.9)
PNC^a^	—	—	1.9 (1.9, 2.1)	1.0 (1.0, 1.1)	0.7 (0.6, 0.7)

^a^ PNC data were not recorded in 1996 and 2001.

At provincial level (year 2016), inequality is largest for ID service use, both for SII and RII (Supplementary File, Supplementary Table S1). The indices are largest for the Karnali Province, followed by the Gandaki Province and the Bagmati province. Sudurpaschim and Lumbini provinces are found to have relatively lower levels of inequality.

### Geographical Variation in the Utilization of Maternal Health Service

**FIGURE 2:**
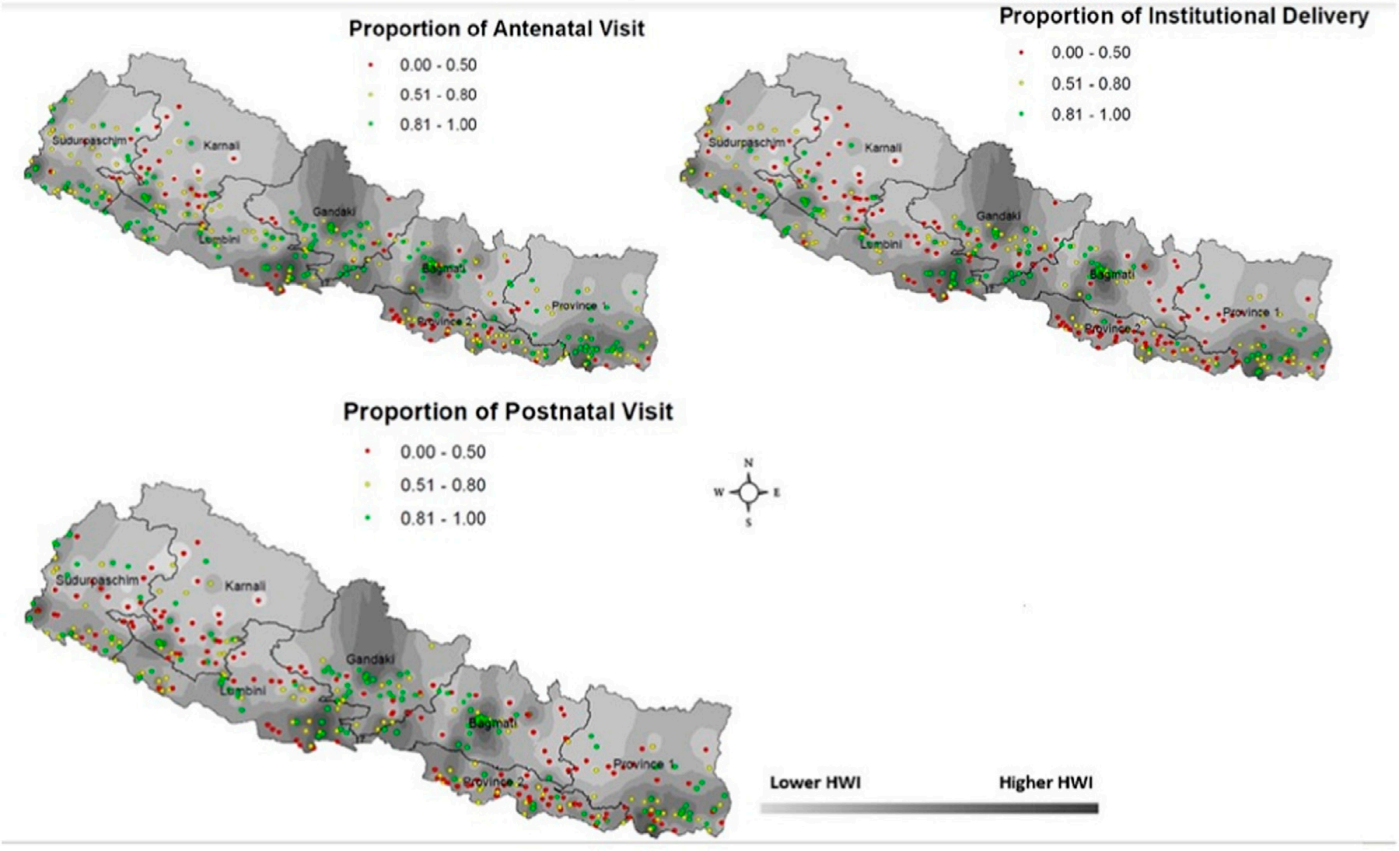
Cluster-level utilization of maternal health services (dots) overlaid on the map of household wealth index (HWI), based on Nepal DHS 2016.

Nepal has seven provinces and 384 geographical clusters (aligned with DHS sampling units). To understand inequality at a granular level, we mapped the spatial distribution of service utilization to show areas with low (0%–50%), medium (50%–80%) and high (80% and above) levels of maternal health services use (Figure 2). Each dot in the map represents a geographic cluster. Overall, there are more “low utilization” (red) clusters in the ID map than in the ANC and PNC maps. This is not surprising given that childbirth at home is still common in rural and remote regions [60], even though non-ID deliveries are associated with higher rates of maternal and neonatal complications and mortality.

**FIGURE 3:**
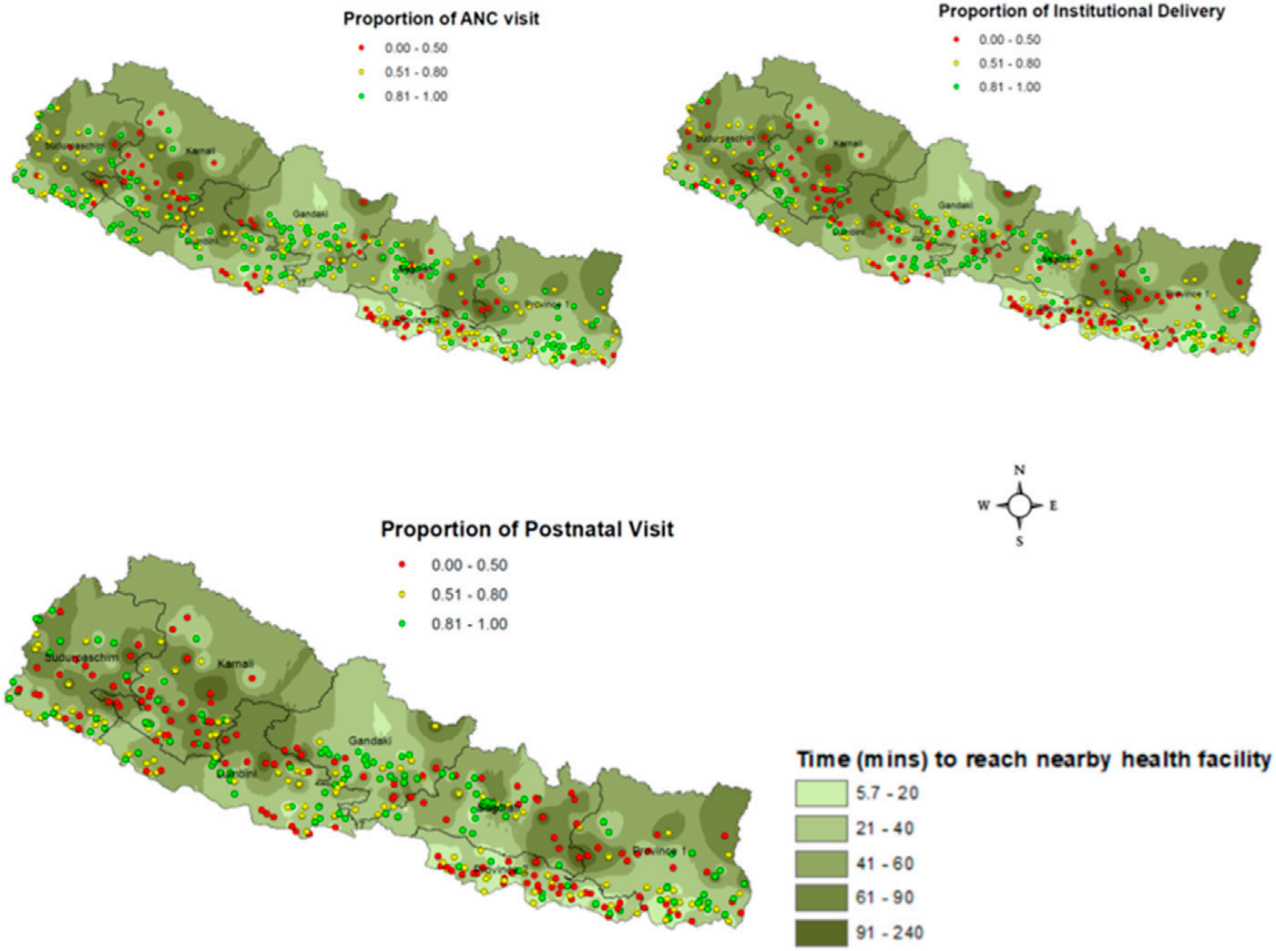
Cluster-level utilization of maternal health services (dots) overlaid on the map of time to travel (in minutes) to the nearest health facility, based on Nepal DHS 2016.

For ANC and PNC, Karnali province was dominated by low utilization (red) clusters (Figure 2). Karnali has mountainous terrain, and travel time to health services is relatively large (Figure 3), often requiring women to walk for hours to reach the nearest health facility [61]. Within this province, the western clusters (bordering Sudurpashchim province) had relatively higher concentration of low utilization clusters because of the challenging terrain and the supply side constraints (i.e., availability of health practitioners).

The other province with low levels of ANC and PNC utilization is Province 2; while this province is plain, it is one of the most resource-deprived provinces of Nepal. This is also the most densely populated province of Nepal, with 630 people/KM^2^ (21% of the population) compared to 61 people/KM^2^ in Karnali province (6% of the population). Province 2 receives the lowest *per capita* budget allocation across all provinces [62] which may partly explain low levels of access to ANC.

The ID map (Figure 2) shows clusters of low service use across the country, except Gandaki province, which has one of the highest *per capita* budget allocation in the country, and the Western part of Bagmati province. This is consistent with the published literature which shows that the geographic distribution of public healthcare facilities is relatively dense in these provinces [61]).

Figure 2 also shows the association between HWI (an indicator of socioeconomic status) and the distribution of clusters. In general, lighter (low wealth) regions tend to have higher concentration of low (red and yellow) clusters. While this is not a deterministic relationship, a pattern can be observed both between and within provinces. For instance, an association between HWI and service use can be seen within Province 1, Bagmati, Gandaki and Lumbini; however, there are exceptions that may be explained by individual-level factors (such as education) and the geographic distribution of health facilities. This spatial map is particularly relevant to health system decision-making as it identifies geographic areas where access to services is limited.

### Decomposing Inequality

This analysis partitions the gap between the richest and poorest socioeconomic quintiles into explained and unexplained portions. Overall, six variables explained the observed gap in service use: parity, mother’s age, mother’s education, father’s education, time to reach the nearby health facility (in minutes) and the caste/ethnicity. These factors explained 43% of the socioeconomic gap for antenatal care, 44% for ID and 42% for PNC (Supplementary File, Table 3).

**TABLE 3:** Decomposition analysis of socioeconomic inequality in utilization of maternal health services Nepal (2016).

	ANC	ID	PNC
Total explained inequality (%)	42.8%	44.0%	42.4%
Contribution of factors as percentage of explained inequality			
Variables	ANC^a^	ID	PNC
Age of the mother	0.2%	0.2%	5.8%
Educational status of the mother	35.7%^b^	22.7%^b^	20.1%^b^
Educational status of the partner	7.9%	4.5%^b^	1.4%
Parity	35.7%^b^	40.9%^b^	31.4%^b^
Time to reach nearest health facility	21.4%^b^	31.8%^b^	42.8%^b^
Caste/ethnicity	0.7%	0.2%	1.4%^b^

^a^ Percentages may not add up to 100 due to rounding.

^b^ Implies statistical significance at 5%.

At national-level, parity explained 35.7% (for ANC), 40.9% (for ID) and 31.4% (for PNC) of the socioeconomic gap. This implies that multiparous women in the lowest socioeconomic group were less likely to use maternal health services than the highest socioeconomic group. Possible explanation is that mothers with previous pregnancies in the poorest quintile may face greater access challenges (e.g., due to childcare), or they rely on their previous experience to manage the current pregnancy. Mother’s education explained 35.7% of the ANC utilization gap, 22.7% of the ID and 20.1% of the PNC gap. Time to reach the nearest health facility explained 21.4% of the socioeconomic gap in ANC, 31.8% in ID and 42.8% in PNC services. Again, this is not surprising since health services in Nepal tend to be concentrated in more populous, urban areas where travel time to health facilities is relatively small. Finally, father’s lack of formal education explained only a small proportion of socioeconomic gap (7.9% for ANC, 4.5% for ID and 1.8% for PNC).

At provincial level, parity, maternal education, caste/ethnicity and travel time to the nearest health facility are the key determinants of inequality, although their relative contribution varied across provinces (Supplementary Figure S1). Maternal education is an important contributor to inequality in province 2 and Karnali. These two provinces have the lowest literacy rates in Nepal. The time to reach nearest health facility has a bigger role in the northern provinces (Province 1, Bagmati, Gandaki, Karnali and Sudurpaschim) that have difficult terrain and sparsely distributed health facilities.

Travel time to health facility can be reduced by investing in new health facilities in areas with low access to services, training and recruiting health professionals in these areas and developing community-based services. Given policy-amenability of this factor, we produced spatial maps to identify clusters with low service use and large travel time to the nearest health facility (Figure 3). This association is particularly strong for Northern provinces where travel time is large and service utilization is low because of poor concentration of health facilities. For instance, the travel time in Karnali province is higher than other provinces—this is associated with higher concentrations of red (low utilization) clusters. This association can also be observed within provinces. For instance, within Gandaki and Bagmati provinces, low use ID clusters are more concentrated in darker (higher travel time) regions. This relationship is not surprising given the geographic distribution of public healthcare facilities in larger rural and urban centres. It should, however, be noted that travel time is only one of the explanatory factors among a host of access barriers.

## Discussion

Nepal experienced a significant increase in the utilization of ANC, ID and PNC between 1996 and 2016. This is largely due to an increase in investment in maternal health services, in line with the priorities identified in Nepal’s Second National Health Plan (1997–2017) [62]. These included interventions such as the Aama Program or safe motherhood program which include free maternal healthcare services and financial incentives for service use [63]. Incentives include cash payments on completion of four ANC visits, ID and PNC visits. In addition, transport incentives of 500–1,500 Nepalese rupees (NPR) are provided for institutional delivery to residents of remote areas, and service incentives are provided to health worker supporting ID. Health facilities also receive cash payments based on the facility size and type of delivery (i.e. 1,000–1,500 NPR for normal delivery, 3,000 for complicated delivery and 7,000 for C-section). These interventions have been found to be effective in resource-poor settings [64, 65].

Despite an increase in average utilization of maternal health services, the socioeconomic gap in maternal health services has persisted over two decades. This is partly because, while maternal health services may be free at the point of delivery in public facilities, indirect out-of-pocket costs can be prohibitively large for poor socioeconomic groups. This is particularly the case for ID services which are often associated with treatment costs that are not covered by public facilities [49]. This may contribute to larger inequities observed in ID relative to ANC and PNC [66]. In addition, safe motherhood programs, such as the National Safe Delivery Incentive Program, that offer cash incentives for institutional delivery have disproportionately higher uptake among wealthier households [66]. This is partly because of the geographic location of healthcare facilities in less deprived areas and the travel-time disparities that result in disproportionately higher benefit incidence of incentive programs in wealthier clusters. This aligns with our finding that Northern provinces (with low density of healthcare facilities) have the highest socioeconomic inequality, both in absolute and relative terms. Parity, maternal education, and travel time to the nearby healthcare facility are important explanatory factors of this socioeconomic gap.

Socioeconomic gap in the utilization of maternal health services has been documented in a number of studies, with South Asian countries reporting some of the highest levels of inequality [67]. The current study found the largest socioeconomic inequality was in the utilization of IDs (56%) due to Nepal’s mountainous terrain, followed by PNC (39%) and ANC (36%). These findings are comparable to other studies in the literature. For instance, a national study in India showed that wealth-based inequality in ANC was 55%, ID was 42% and post-natal care was 38% [68]. Similarly, a study from Mali found a socioeconomic inequity gap of 66% for ID and 54% for ANC (54%) [69]. In Ethiopia, the socioeconomic gap was 46% for ID, 22% for ANC and 9% for PNC [70].

Identification of the determinants of inequalities can help policymakers narrow the socioeconomic gap. We found that, at the national level, 43% of the socioeconomic gap in the ANC, 45% in ID and 33% in PNC could be explained by six variables, i.e., age of the mother, parity, educational status of the father, educational status of mother, caste/ethnicity, and travel time to nearest healthcare facility. Travel time plays a significant role in the highland provinces (Gandaki, Karnali, Bagmati, and Sudurpaschim) while parity and maternal education are bigger contributors in Province 2 and 1.

Previous studies have reported that poor education of parents (particularly mothers), parity and ethnicity/caste are important determinants of inequity in maternal health services use [41, 71–77]. For instance, a study from Pakistan reported that nulliparous women were 4.1 times more likely to visit ANC compared to multiparous women [75]. This could be due to a number of factors including the need to take care of children at home [72] and reliance on previous childbearing experience to help with the current pregnancy [76].

Time to reach the nearest health facility is another significant predictor of healthcare utilization, particularly affecting deprived rural population [71, 78, 79]. Our study makes a unique contribution by spatially mapping those areas where travel time to the nearest facility is large; this provides actionable intelligence to policymakers to guide resource allocation decisions. More specifically, the combination of spatial data on travel time and clusters of low service use will help decision-makers identify specific local areas where new health facilities can be built, and community-based programs can be implemented to improve geographic access to services. This strengthens previous spatial analyses of potential new health facility locations in Nepal [61].

The significance of the role of travel time to health facility should be interpreted in the light of topography. For instance, for province 2, which is relatively non-mountainous and has a high population density, the time to reach the health facility makes only a small contribution to explaining the socioeconomic gap. Our findings are consistent with Cao et al [61] who mapped travel-time disparities in access to public healthcare facilities for all services in Nepal and found that disparities are worse in the northern mountain belt in Nepal, both in terms of walking and motorized travel modes.

This study has some limitations. First, the relationships identified in the analysis are associational and not causal as the study is based on cross-sectional data. Second, there was no data on the quality of maternal health services received by respondents—this can be important because disadvantaged groups may receive suboptimal care compared to those with access to better quality care. Third, the survey questionnaire did not ask about the number of postnatal visits but only included questions related to the first visit. As a result, we could not assess inequality in the use of at least 3 visits, as recommended by the WHO. Fourth, data on travel time to the nearest health facility may not take into account mobile health clinics. Finally, our analysis could be subjected to recall and reporting bias.

### Conclusion

While the average rate of maternal health service use in Nepal has increased over time, the socioeconomic inequity has persisted over 20 years. Maternal education and time to travel to the nearest health facility are the key factors that explain these inequities; however, the contribution of these factors varied across provinces. This study also provides spatial maps to help decision-makers identify regions where inequities exists.
