# Supplementary material for: Investigating Health Inequality Using Trend, Decomposition and Spatial Analyses: A Study of Maternal Health Service Use in Nepal
Source: Int J Public Health. 2023 Jun 2;68:1605457. doi: 10.3389/ijph.2023.1605457 (PMC10272384; doi:10.3389/ijph.2023.1605457)

**SUPPLEMENTARY MATERIAL**

**Table S1:** Provincial-level slope and relative indices of inequality (NDHS- 2016*)

| **Provinces** | **Antenatal Care visit** | | **Institutional delivery** | | **Postnatal care Visit** | |
| --- | --- | --- | --- | --- | --- | --- |
|  | **SII**  **(95% CI)** | **RII**  **(95% CI)** | **SII**  **(95% CI)** | **RII**  **(95% CI)** | **SII**  **(95% CI)** | **RII**  **(95% CI)** |
|  |  |  |  |  |  |  |
| Province 1 | 29.3  (28.7, 30.0) | 0.38  (0.37, 0.39) | 65.42  (64.05, 66.79) | 1.06  (1.04, 1.08) | 35.79  (35.37, 36.22) | 0.59  (0.58, 0.60) |
| Province 2 | 27.39  (27.30, 27.47) | 0.49  (0.493, 0.50) | 33.2  (33.03, 33.39) | 0.8  (0.79, 0.80) | 26.91  (26.84, 26.98) | 0.56  (0.56, 0.57) |
| Bagmati | 46.95  (45.01, 48.90) | 0.61  (0.58, 0.63) | 81.5  (77.63, 85.39) | 1.22  (1.16, 1.28) | 59.05  (56.67, 61.44) | 0.89  (0.85, 0.93) |
| Gandaki | 57.21  (54.46, 59.96) | 0.75  (0.71, 0.79) | 75.70  (72.46, 78.94) | 1.12  (1.07, 1.17) | 68.60  (65.46, 71.75) | 1.05  (1.00, 1.10) |
| Lumbini | 21.50  (21.24, 21.76) | 0.29  (0.28, 0.29) | 51.78  (51.14, 52.41) | 0.86  (0.85, 0.87) | 18.71  (18.61, 18.82) | 0.30  (0.31, 0.39) |
| Karnali | 65.0  (63.82, 66.14) | 1.09  (1.07, 1.11) | 74.39  (73.02, 75.75) | 1.54  (1.51, 1.57) | 65.41  (64.17, 66.65) | 1.34  (1.31, 1.37) |
| Sudur Paschim | 21.63  (21.29, 21.97) | 0.28  (0.28, 0.29) | 38.45  (37.89, 39.00) | 0.58  (0.57, 0.588) | 16.21  (16.13, 16.29) | 0.27  (0.27, 0.27) |

* Provincial-level indices are computed only for the latest wave, i.e. 2016, because provinces were established in 2015.

**Fig S1:** Contribution of factors explaining inequity in utilization of maternal health services across provinces in Nepal, DHS-2016


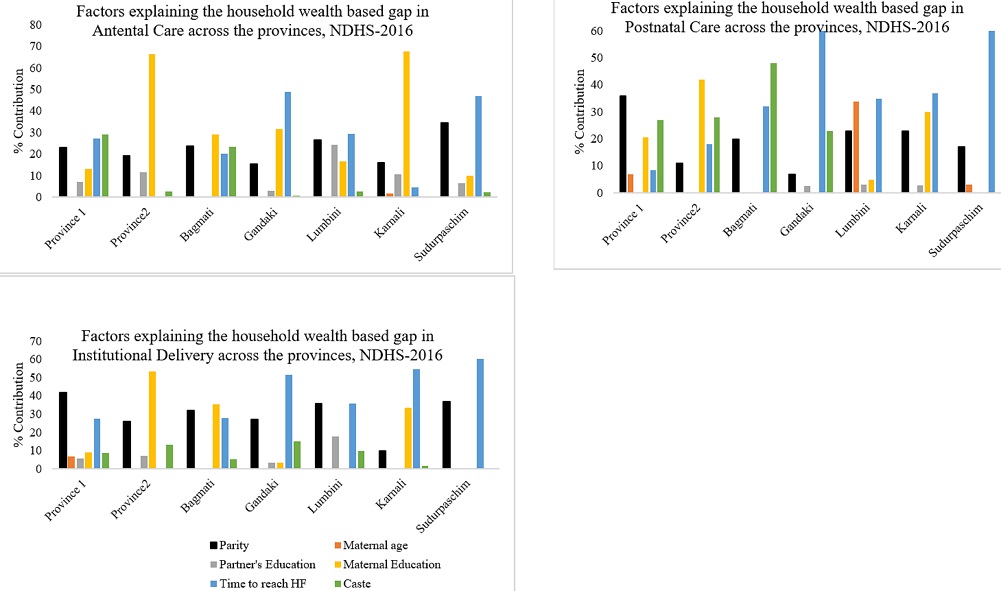

Supplement: Supplementary file 1 [file DataSheet1.docx]
